# Supplementary material for: Ex vivo cortical circuits learn to predict and spontaneously replay temporal patterns
Source: Nat Commun. 2025 Apr 4;16:3179. doi: 10.1038/s41467-025-58013-z (PMC11971321; doi:10.1038/s41467-025-58013-z)
Supplement: Supplementary file 1 — Supplementary Information [file 41467_2025_58013_MOESM1_ESM.pdf]

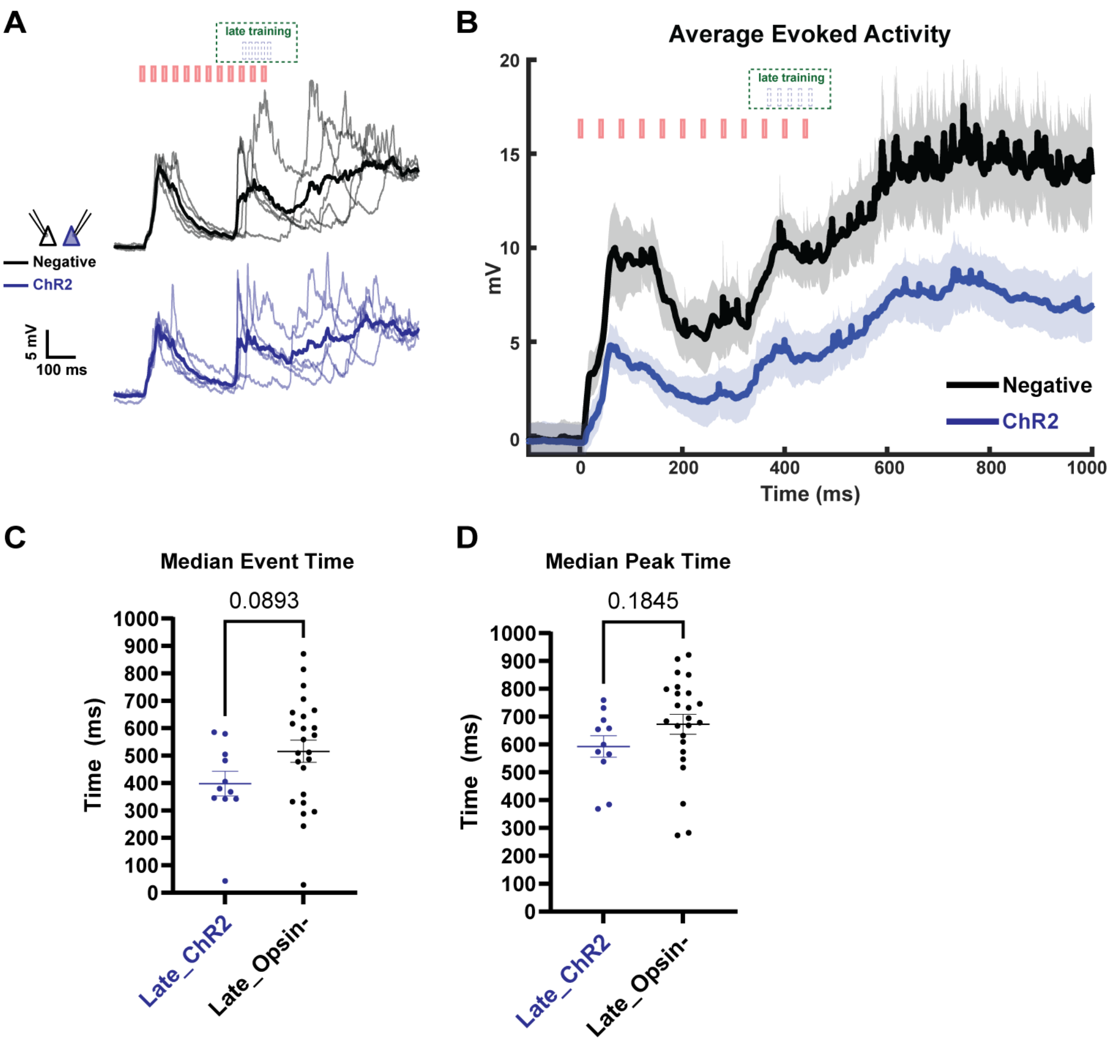

Supplementary Figure 1. Following Late-training the temporal profile of evoked activity was not significantly different between simultaneously recorded ChR2$^+$ and opsin-negative neurons. A, Sample traces of evoked polysynaptic activity (5 traces per cell and mean (bold)) from a simultaneously recorded pair of ChR2$^+$ and Opsin$^-$ pyramidal neurons in response to red-alone. **B,** Comparison of the mean ± SEM (shading) of evoked activity in simultaneously recorded ChR2$^+$ and Opsin$^-$ pyramidal neurons from Late-trained slices (Opsin$^-$: 8, ChR2$^+$: 8). **C,** Average median event time of evoked network activity was not significantly different in ChR2$^+$ and Opsin$^-$ neurons from Late trained slices. Data are presented as median event times ± SEM: 398 ± 45 ms and 516 ± 41 ms for ChR2$^+$ and Opsin$^-$ pyramidal neurons, respectively; n = 11, n = 24, p = 0.089, two-sided unpaired t-test. **D,** Average median peak time of evoked network activity was not significantly different in ChR2$^+$ and Opsin$^-$ neurons from Late trained slices. Data are presented as median peak times ± SEM: 593 ± 38 ms and 673 ± 36 ms for ChR2$^+$ and Opsin$^-$ pyramidal neurons, respectively; n = 11, n = 24, p = 0.185, two-sided unpaired t-test.
