# Supplementary material for: Ex vivo cortical circuits learn to predict and spontaneously replay temporal patterns
Source: Nat Commun. 2025 Apr 4;16:3179. doi: 10.1038/s41467-025-58013-z (PMC11971321; doi:10.1038/s41467-025-58013-z)
Supplement: Supplementary file 2 — Reporting Summary [file 41467_2025_58013_MOESM2_ESM.pdf]

nature portfolio

Corresponding author(s): Dean Buonomano

Last updated by author(s): Dec 4, 2024

Reporting Summary

Nature Portfolio wishes to improve the reproducibility of the work that we publish. This form provides structure for consistency and transparency in reporting. For further information on Nature Portfolio policies, see our Editorial Policies and the Editorial Policy Checklist.

Statistics

For all statistical analyses, confirm that the following items are present in the figure legend, table legend, main text, or Methods section.

n/a	Confirmed	
☐	☒	The exact sample size (n) for each experimental group/condition, given as a discrete number and unit of measurement
☐	☒	A statement on whether measurements were taken from distinct samples or whether the same sample was measured repeatedly
☐	☒	The statistical test(s) used AND whether they are one- or two-sided<br>*Only common tests should be described solely by name; describe more complex techniques in the Methods section.*
☐	☒	A description of all covariates tested
☐	☒	A description of any assumptions or corrections, such as tests of normality and adjustment for multiple comparisons
☐	☒	A full description of the statistical parameters including central tendency (e.g. means) or other basic estimates (e.g. regression coefficient) AND variation (e.g. standard deviation) or associated estimates of uncertainty (e.g. confidence intervals)
☐	☒	For null hypothesis testing, the test statistic (e.g. F, t, r) with confidence intervals, effect sizes, degrees of freedom and P value noted<br>*Give P values as exact values whenever suitable.*
☒	☐	For Bayesian analysis, information on the choice of priors and Markov chain Monte Carlo settings
☒	☐	For hierarchical and complex designs, identification of the appropriate level for tests and full reporting of outcomes
☐	☒	Estimates of effect sizes (e.g. Cohen's d, Pearson's r), indicating how they were calculated

Our web collection on statistics for biologists contains articles on many of the points above.

Software and code

Policy information about availability of computer code

Data collection	Data collection was done via Signal 7 software version 7.07 from Cambridge Electronic Design.
Data analysis	Data analysis was done via custom written MATLAB code which were made available on the OSF database: https://osf.io/qspnv/

For manuscripts utilizing custom algorithms or software that are central to the research but not yet described in published literature, software must be made available to editors and reviewers. We strongly encourage code deposition in a community repository (e.g. GitHub). See the Nature Portfolio guidelines for submitting code & software for further information.

Data

Policy information about availability of data

All manuscripts must include a data availability statement. This statement should provide the following information, where applicable:

- Accession codes, unique identifiers, or web links for publicly available datasets
- A description of any restrictions on data availability
- For clinical datasets or third party data, please ensure that the statement adheres to our policy

The data generated from patch-clamp recordings which represent the central findings in this study have been deposited in The Open Science Framework database: https://osf.io/qspnv/. Source data for figures 2-6 are provided with this paper in the Source Data file.

Research involving human participants, their data, or biological material

Policy information about studies with human participants or human data. See also policy information about sex, gender (identity/presentation), and sexual orientation and race, ethnicity and racism.

Reporting on sex and gender	N/A
Reporting on race, ethnicity, or other socially relevant groupings	N/A
Population characteristics	N/A
Recruitment	N/A
Ethics oversight	N/A

Note that full information on the approval of the study protocol must also be provided in the manuscript.

Field-specific reporting

Please select the one below that is the best fit for your research. If you are not sure, read the appropriate sections before making your selection.

[x] Life sciences [ ] Behavioural & social sciences [ ] Ecological, evolutionary & environmental sciences

For a reference copy of the document with all sections, see nature.com/documents/nr-reporting-summary-flat.pdf

Life sciences study design

All studies must disclose on these points even when the disclosure is negative.

Sample size	No statistical method was used to predetermine sample size for the study. While collecting data and performing significance tests we considered the desired confidence interval and margin of error to ensure the reliability and precision of data.
Data exclusions	The only data that was excluded was from poor electrophysiological recordings (in vitro whole-cell patch clamp), data was excluded based on pre-defined criteria from previous publications in the lab (e.g. resting membrane potential above -50mV, drifting voltage).
Replication	Replications were successful, seven batches of organotypic slices were prepared from different mice at different time periods over the course of a year and trained in the same conditions. Recordings from these different batches yielded consistent results. A different method of recording outside the scope of this study was also used in the lab by another lab member with success in replicating the results.
Randomization	Organotypic slices were prepared from mice without discrimination of sex, slices were treated equally and trained without bias. Control slices (untrained slices) came from the same mice as the trained slices without bias.
Blinding	Investigators were not blind to the training condition during data collection, since the investigator would start and stop the specific types of training for each organotypic slice before recording from the slice on the rig. Investigators were blind to the training condition during analysis of the data.

Reporting for specific materials, systems and methods

We require information from authors about some types of materials, experimental systems and methods used in many studies. Here, indicate whether each material, system or method listed is relevant to your study. If you are not sure if a list item applies to your research, read the appropriate section before selecting a response.

Materials & experimental systems

n/a	Involved in the study	
[x]	[ ]	Antibodies
[x]	[ ]	Eukaryotic cell lines
[x]	[ ]	Palaeontology and archaeology
[ ]	[x]	Animals and other organisms
[x]	[ ]	Clinical data
[x]	[ ]	Dual use research of concern
[x]	[ ]	Plants

Methods

n/a	Involved in the study	
[x]	[ ]	ChIP-seq
[x]	[ ]	Flow cytometry
[x]	[ ]	MRI-based neuroimaging

Animals and other research organisms

Policy information about studies involving animals; ARRIVE guidelines recommended for reporting animal research, and Sex and Gender in Research

Laboratory animals	Wildtype FVB mice of either sex, between ages of Postnatal day 5 - 7
Wild animals	N/A
Reporting on sex	Sex was not considered in the study design, however equal amounts of mice of either sex were used in the study.
Field-collected samples	N/A
Ethics oversight	Animal ethics guidance and research protocol approval was provided by the UCLA Animal Research Committee

Note that full information on the approval of the study protocol must also be provided in the manuscript.

Plants

Seed stocks	N/A
Novel plant genotypes	N/A
Authentication	N/A
